# Взаимозаменяемость различных косвенных методов определения состава тела

**DOI:** 10.14341/probl13538

**Published:** 2025-09-14

**Authors:** Э. А. Бондарева, Б. А. Гарасько, Н. Н. Хромов-Борисов, Н. В. Мазурина, Е. В. Ершова, К. А. Комшилова, Е. А. Трошина

**Affiliations:** Федеральный научно-клинический центр физико-химической медицины им. акад. Ю.М. Лопухина Федерального медико-биологического агентства; Lopukhin Federal research and clinical center of physical-chemical medicine, Federal medical biological agency; Комиссия РАН по борьбе с лженаукой; Commission on Pseudoscience of Russian Academy of Sciences; Национальный медицинский исследовательский центр эндокринологии им. акад. И.И. Дедова; I.I. Dedov Endocrinology Research Centre

**Keywords:** биоимпедансометрия, состав тела, жировая масса, ультразвуковое сканирование, InBody, BodyMetrix, BIA, body composition, fat mass, ultrasound scanning, InBody, BodyMetrix

## Abstract

**BACKGROUND:**

BACKGROUND: Determination of body composition components — muscle and fat mass — is an important step in clinical and epidemiological studies. The most common methods for quantitative determination of body composition are indirect methods. However, the variety of methods and models of devices used makes direct comparison of data at both group and individual levels difficult.

**AIM:**

AIM: The aim of the study is to analyze the consistency of estimates of absolute values of fat and lean body mass, as well as the proportion of body fat mass, obtained using bioimpedance analyzers ABC-02 «Medas» (STC Medas, Russia), 770InBody (InBody, Korea) and ultrasound scanner BodyMetrix BX2000 (IntelaMetrix, USA) in a group of men and women.

**MATERIALS AND METHODS:**

MATERIALS AND METHODS: An observational, single-center, cross-sectional, uncontrolled study was conducted. The main anthropometric characteristics (height and weight, waist circumference) were measured. Body composition was determined by bioimpedancemetry (BIA) using the octopolar scheme on the 770InBody device and the tetrapolar scheme on the ABC-02 Medass device and ultrasound scanning using the BodyMetrix BX2000 (BM) ultrasound scanner. The absolute (FM) and relative amount of body fat (PBF) and lean body mass were calculated.

**RESULTS:**

RESULTS: A total of 48 people (38 women and 10 men) aged 24 to 74 years were examined. The anthropometric characteristics of the examined subjects were presented in a wide range. A strong correlation was found for all pairs of body composition components: the minimum value for the pair PBF ABC-BM was 0.853 [0.730, 0.913], the maximum was 0.988 [0.977, 0.993] for the pair FM ABC-InBody. Also, significant statistical differences (p<0.001) were found for all pairs of measurements, except for PBF determined by the BIA method. High agreement (CCC>0.95) of BIA estimates of the absolute amount of fat mass was shown, moderate agreement (CCC 0.9–0.95) is characteristic of the PBF determined by different BIA analyzers, and for all other pairs the agreement of measurements can be assessed as weak (CCC<0.90).

**CONCLUSION:**

CONCLUSION: The best agreement at the group and individual levels was found for FM estimates by two different BIA analyzers (InBody and ABC).

## ОБОСНОВАНИЕ

Определение компонентов состава тела необходимо в медицинских целях для оценки количества мышечной массы тела у пожилых и тяжелобольных пациентов, в популяционных исследованиях распространенности ожирения, для оценки индивидуального риска развития коморбидных ожирению или саркопении заболеваний, в практике подготовки спортсменов, а также в антропологических исследованиях различных популяций современного человека [1]. В связи с перечисленными причинами для диагностики нутритивного статуса в медицинских исследованиях широко применяются методы, позволяющие количественно определить состав тела [1]. Среди значительного разнообразия данных методов можно выделить крайне мощные и точные референсные (эталонные) методы (подводное взвешивание, воздушнозаместительную плетизмографию, нейтронно-активационный анализ, двухэнергетическую рентгеновскую денситометрию, методы компьютерной томографии) и косвенные или полевые методы (калиперометрия, биоимпедансометрия, ультразвуковое сканирование, расчет по аналитическим формулам из простых антропометрических признаков) [1][2]. Косвенные методы обладают меньшей точностью, однако они получили значительно более широкое распространение в связи с быстротой и низкой стоимостью процедур, отсутствием потенциально вредных воздействий на организм, мобильностью, а также они могут быть использованы в широком возрастном диапазоне [3]. Биоимпедансный анализ предполагает соблюдение ряда допущений и условий: отсутствие кардиостимулятора и/или металлических имплантов, обследование натощак, отсутствие физической нагрузки накануне и непосредственно перед исследованием, запрет на употребление алкоголя накануне. Кроме того, факторы, которые изменяют гидратацию тканей (заболевания, прием некоторых препаратов, питьевой режим), искажают оценки состава тела [4]. Эти условия в ряде случаев накладывают ограничения на использование биоимпедансометрии. Прямое сопоставление данных, получаемых с использованием приборов биоимпедансного анализа (БИА-приборов) от разных производителей, затруднено, так как уравнения для расчета компонента состава тела из показателей активного сопротивления не всегда приводятся производителем в открытых источниках или инструкциях к поставляемому оборудованию. Это, с одной стороны, ограничивает применение БИА в эпидемиологических и клинических исследованиях, а с другой — затрудняет стандартизацию и сопоставление данных, полученных на разных биоимпедансных анализаторах.

Ультразвук был предложен как альтернатива измерению толщины кожно-жировых складок, так как он лишен недостатков калиперометрии. К недостаткам расчета массы жировой ткани по результатам измерения толщин кожно-жировых складок калипером относятся следующие: значительная вариабельность результатов в зависимости от квалификации исследователя, сложность или невозможность определения границы подкожная жировая клетчатка-мышца, влияние уровня гидратации кожи и тканей, невозможность корректного измерения у людей с ожирением, связанная с ограниченной шириной, на которую могут быть разведены лапки калипера, распределение соединительной ткани и кровеносных сосудов, которое может исказить толщину измеряемой кожно-жировой складки [5–7]. В то же время УЗИ позволяет одновременно проводить оценку толщины скелетной мышцы в месте измерения [8]. Ультразвуковые сканеры могут измерять толщину подкожного жира на 100 и более мм без сдавливания тканей и определять границы раздела между тканями с точностью 1 мм. В 1960–1970 гг. было показано, что ультразвуковые измерения толщины подкожного жира тесно коррелируют с прямыми измерениями подкожного жира (r=0,98) посредством электропроводимости [8–9]. Особенности оценки состава тела методом ультразвукового исследования собраны и систематизированы в современных работах [5–7][10].

## ЦЕЛЬ ИССЛЕДОВАНИЯ

Целью исследования является анализ согласованности оценок абсолютных значений жировой и безжировой массы тела, а также доли жировой массы тела, полученных с применением биоимпедансных анализаторов АВС-02 «Медасс», 770InBody и ультразвукового сканера BodyMetrixTM (IntelaMetrix, США) в группе мужчин и женщин.

## МАТЕРИАЛЫ И МЕТОДЫ

## Место и время проведения исследования

Место проведения. Национальный медицинский исследовательский центр эндокринологии, Москва.

Время исследования. Июнь–июль 2024 г.

## Изучаемые популяции (одна или несколько)

Критерии включения: мужчины и женщины старше 17 лет.

Критерии исключения: наличие металлических имплантов, наличие кардиостимулятора, беременность и период лактации.

## Способ формирования выборки из изучаемой популяции (или нескольких выборок из нескольких изучаемых популяций)

Сплошной (пациенты, поступавшие на стационарное лечение).

## Дизайн исследования

Проведено обсервационное одноцентровое одномоментное неконтролируемое исследование.

## Методы

Антропометрическое обследование включало измерение длины тела (ДТ) лазерным антропометром (КАФА, Россия), обхватов талии (ОТ) и бедер (ОБ) неэластичной измерительной лентой и массы тела (МТ) (770InBody). Было проведено определение состава тела методами биоимпедансометрии (АВС-02 «Медасс» и 770InBody) и ультразвукового сканера BodyMetrix BX2000 (IntelaMetrix, США). Здесь и далее для них использованы сокращенные обозначения: ABC, IB и BM соответственно.

БИА выполняли с применением АВС-02 «Медасс» (НТЦ «Медасс»; Россия) по стандартной тетраполярной схеме «запястье-голеностопный сустав» на правой стороне тела с наложением электродов F3001 (FIAB; Италия) при положении испытуемых лежа на спине.

БИА на анализаторе InBody 770 (Корея) выполняли согласно протоколу производителя. Были определены абсолютные и относительные значения жировой (ЖМ и %ЖМ соответственно), скелетно-мышечной массы (СММ).

Ультразвуковое определение состава тела проводили с применением сканера BodyMetrix BX2000 (IntelaMetrix, США) в положении обследованного стоя [11]. Использовали формулу Джексона-Поллока для семи точек. В качестве контактной среды был использован гель для ультразвуковых исследований средней вязкости «Медиагель» («Гельтек»; Россия).

## Статистический анализ

В статистике нет единого универсального метода проверки согласованности лабораторных методов. Поэтому согласно современным рекомендациям, для решения основной задачи настоящего исследования — оценить согласованность трех косвенных методов определения состава тела — в работе использовали комплексный подход [12][13] с вычислением информационно взаимодополняющих статистических показателей: средняя разность (MD); стандартизированная средняя разность (размер эффекта по Коэну для парных наблюдений с учетом их корреляции, dRM) [14]; коэффициенты корреляции Пирсона (r) и Спирмена (rS); коэффициент конкордатной корреляции Лина (CCC), который измеряет согласие между двумя измерениями одной и той же переменной, и его рекомендуют для оценки воспроизводимости при повторении экспериментов, согласия между результатами разных методов и т.п., анализ Блэнда-Олтмена.

В подавляющем большинстве случаев использовали алгоритмы бутстрепа, как наиболее надежные (непараметрические) и предпочтительные для задач статистического оценивания параметров большинства типов данных (вне зависимости, например, от формы распределения) [16]. Для этого использовали программы PAST, JASP и jamovi [15]. Для вычисления размера эффекта с учетом корреляции использовали пакет «statpsych» в среде R.

Согласно современным рекомендациям, для всех оцениваемых в работе показателей вычисляли 95%-е ДИ или бейзовские заслуживающие внимания (credible) интервалы.

Там, где было возможно, использовали современные методы бейзовской статистики. В частности, для коэффициентов корреляции использовали медиану их апостериорных распределений и 95%-е ДИ. В качестве интегрального показателя для сравнения правдоподобий нулевой и альтернативной гипотез использовали бейзовы факторы: BF10 — в пользу альтернативной гипотезы против нулевой, или BF01 — в пользу нулевой гипотезы против альтернативной. Согласно общепринятой договоренности, существенными принимали эффекты с BF10>100. Значения p указывали исключительно как дань традиции. Согласно современным рекомендациям статистически значимыми признавали эффекты с p<0,005 (а не 0,05).

Заслуживающими внимания считали размеры эффекта, для которых нижняя граница 95%-х ДИ превышала значение dRM=1 [17]; корреляции, для которых нижняя граница 95%-х ДИ превышала значение r=0,8; конкордатную корреляцию, для которой нижняя граница 95%-х ДИ превышала значение ССС=0,95.

Для анализа Бленда-Олтмена и для точечной и интервальной оценок ССС использовали модуль Simple Agreement Analysis из пакета jamovi и https://huygens.science.uva.nl/BA-plotteR/. Анализ эквивалентности проводили в пакете «TOSTER». Для контроля ошибки первого рода при множественных попарных сравнениях использовали поправку Холма.

## Этическая экспертиза

Локальный этический комитет при ГНЦ ФГБУ «НМИЦ эндокринологии» Минздрава России постановил одобрить возможность проведения данной научно-исследовательской работы в рамках Государственного задания «Механизмы дезадаптации двухуровневой системы регуляции аппетита при экзогенно-конституциональном ожирении с множественными осложнениями и способы ее коррекции». Всеми пациентами подписано информированное согласие на участие в исследовании.

## РЕЗУЛЬТАТЫ

Были обследованы 48 человек (38 женщин и 10 мужчин) в возрасте от 24 до 74 лет. В обследованной выборке ИМТ (кг/м²)<18,5 имели три человека, ИМТ 18,5–24,9 — 10 человек, ИМТ 25–29,9 — 2 человека, ИМТ 30–35,7 — 11 человек, ИМТ 36–40 и ИМТ>40 — 15 человек.

Характеристики исследованных признаков приведены в таблице 1. Среди исследованных признаков половой диморфизм обнаружен по длине тела, доле жировой массы тела, определенной в УЗИ, а также по абсолютным значениям безжировой (УЗИ) и скелетно-мышечной массе тела (БИА).

**Table:** Таблица 1. Значения компонентов состава тела, определенных методами УЗИ и БИА в общей выборке

Признак	Метод	Среднее (M)	Стандартное отклонение (SD)	Коэффициент вариации (CV, %)
Доля жировой массы (%ЖМ), %	BM	35 [ 32; 37]	8,7 [ 6,6; 10,4]	25 [ 18; 31]
InBody	41 [ 38; 45]	12,6 [ 9,9; 14,6]	31 [ 23; 39]
ABC	40 [ 36; 43]	11,7 [ 9,7; 13,6]	30 [ 22; 37]
Жировая масса (ЖМ), кг	BM	36 [ 31; 41]	17,8 [ 15,1; 20,6]	49 [ 29; 59]
InBody	44 [ 37; 51]	24,7 [ 20,9; 28,5]	56 [ 46; 68]
ABC	42 [ 36; 48]	23,2 [ 19,5; 26,8]	55 [ 45; 67]
Безжировая масса (БЖМ), кг	BM	64 [ 57; 70]	22,5 [ 16,3; 27,3]	35 [ 28; 41]
ABC	56 [ 52; 62]	14,6 [ 10,8; 17,7]	26 [ 20; 31]
Скелетно-мышечная масса (СММ), кг	InBody	30 [ 28; 32]	7,6 [ 6,0; 8,9]	25 [ 21; 30]
ABC	25 [ 23; 37]	7,2 [ 5,5; 8,7]	29 [ 24; 34]

Для всех пар компонентов состава тела обнаружена сильная корреляционная связь (табл. 2). Между показателями %ЖМ и ЖМ, определенными данными методами, обнаружены значимые статистические различия. Попарные сравнения выявили статистические различия для всех сравниваемых пар: %ЖМ ABC-BM pholm = 10∙10-7 (BF10 = 77700), %ЖМ ABC- InBody pholm = 0,053 (BF10 = 5,03), %ЖМ InBody -BM pholm = 3∙10-11 (BF10 = 94480), ЖМ ABC-BM pholm = 3∙10-7 (BF10 = 20400), ЖМ ABC- InBody pholm = 0,062 (BF10 = 18), ЖМ InBody -BM pholm = 9∙10-11 (BF10 = 29800).

**Table:** Таблица 2. Попарные сравнения методов и корреляции между ними Примечание: MDRM и dRM — соответственно, средняя разность и стандартизированный размер эффекта с поправкой на коррелированность повторных измерений, ССС — коэффициент конкордантной корреляции Лина. ЖМ — жировая масса, БЖМ — безжировая масса, СММ — скелетно-мышечная масса.

Показатель	Пара	MDRM [ 95%ДИ]	dRM [ 95%ДИ]	CCC [ 95%ДИ]	Точные 90%-е ДИ для критерия Shieh
%ЖМ	BM — InBody	-6,7 [ -8,8; -4,7]	-0,62 [ -0,85; -0,40]	0,67 [ 0,53; 0,77]	-23; 9,5
BM — ABC	-5;0 [ -6,6; -3,5]	-0,48 [ -0,67; -0,31]	0,78 [ 0,67; 0,83]	-17; 7,3
InBody — ABC	1,7 [ 0,62; 2,8]	0,14 [ 0,048; 0,24]	0,94 [ 0,90; 0,97]	-6,8; 10
ЖМ	BM — InBody	-7,8 [ -11; -5,1]	-0,36 [ -0,50; -0,22]	0,85 [ 0,78; 0,90]	-30; 13
BM — ABC	-5,8 [ -7,8; -3,7]	-0,27 [ -0,39; -0,16]	0,90 [ 0,85; 0,93]	-23; 10
InBody — ABC	2,1 [ 0,89; 3,2]	0,085 [ 0,04; 0,14]	0,98 [ 0,97; 0,99]	-6,9; 11
БЖМ	BM — ABC	7,6 [ 4,8; 11]	0,40 [ 0;23; 0,57]	0,82 [ 0,75; 0,88]	-14; 28
СММ	InBody — ABC	5,1 [ 4,3; 6;0]	0,68 [ 0,51; 0,87]	0,74 [ 0;63; 0,82]	-1,5; 12

Невооруженным глазом видно, что BM слегка занижает значения по сравнению с IB и ABC, которые практически не различаются (рис. 1). Это подтверждают полученные 95%-е для средних разностей и размеров эффекта и р-значения для попарных сравнений. Сравнение размера эффекта в парах исследованных признаков показало, что для %ЖМ в паре БИА, ЖМ для всех пар и БЖМ в паре BM-АВС dc статистически незначим (табл. 2). Для остальных пар dc значимо отличается от нулевого значения, однако область 95%ДИ содержит диапазон слабых значений. То есть размер эффекта с практической точки зрения позволяет считать наблюдаемые различия пренебрежимо малыми, несмотря на формальное преодоление уровня значимости.

**Figure:**
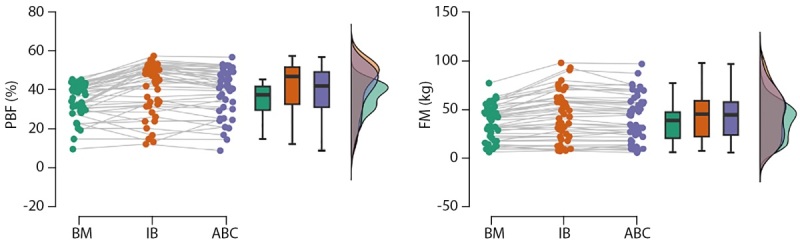
Рисунок 1. Сравнительный анализ показателей %ЖМ (PBF) и ЖМ (FM), определенных в УЗ-сканировании (BM), в октополярной схеме БИА (InBody) и тетраполярной схеме БИА (АВС).

Наглядно корреляции представлены на рисунке 2.

**Figure:**
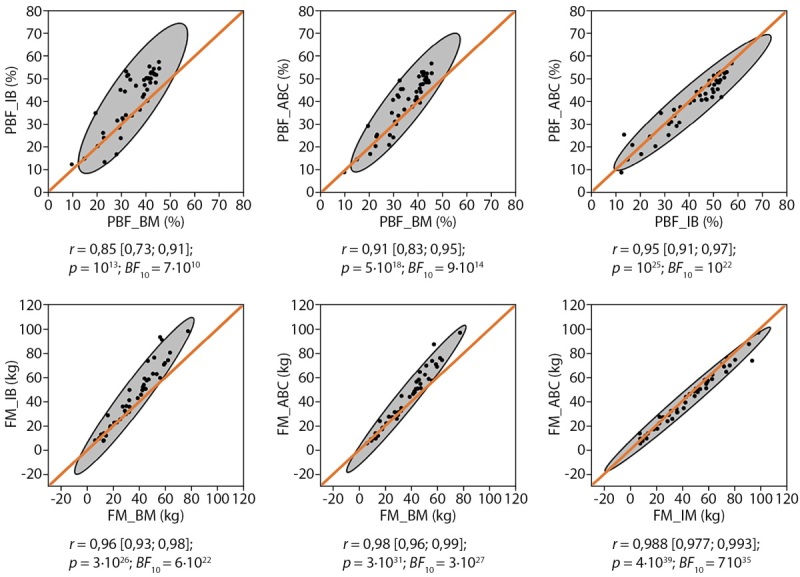
Рисунок 2. Медианы апостериорного распределения коэффициента корреляции Пирсона r c 95%м ДИ. Эллипсы — границы 95%х доверительных зон для диаграмм рассеяния. Диагонали — прямые, отражающие идеальное совпадение результатов сопоставляемых методов (ССС=1).

Анализ позволяет заключить, что высокую согласованность (CCC>0,95) демонстрирует БИА оценка абсолютного количества жировой массы (ЖМ), умеренная согласованность (CCC 0,9–0,95) характерна для доли жировой массы (%ЖМ), определенной разными БИА анализаторами, а для всех остальных пар согласованность измерений можно оценить как слабую (CCC<0,90) (табл. 3). Для всех пар измерений обнаружено смещение оценок, максимальное смещение характерно для ЖМ и %ЖМ в паре BodyMetrix-InBody770, минимальное — для ЖМ и %ЖМ в паре АВС-02-InBody770. Для пары АВС-02-InBody770 выявлены также самые узкие границы согласованности (табл. 3). На графиках Бленда-Альтмана видно, что для пар измерений УЗИ-БИА в области низких значений показателя BodyMetrix дает оценки выше, чем БИА анализаторы, а в области высоких значений — наоборот (рис. 3).

**Table:** Таблица 3. Анализ Блэнда-Олтмена для оценки согласия в парах использованных методов

Параметр	Доля жировой массы; %	Жировая масса; кг
BM-IB	IB-ABC	BM-IB	BM-IB	IB-ABC	BM-IB
Средняя разность	-6,6 [ -8,8; -4,4]	-1,6 [ -3,2; -0,59]	-4,96 [ -6,6; -3,3]	-6,7 [ -11; -3,3]	-1,6 [ -2,9; -0,6]	-4,9 [ -6,9; -1,4]
Верхняя граница согласованности	7,3 [ 3,7; 11]	3,7 [ 1,1; 10]	5,6 [ 2,8; 8,4]	2,5 [ 0,2; 5,2]	2,1 [ 1,0; 6,1]	2,5 [ 0,4; 3,1]
Нижняя граница согласованности	-21 [ -24; -17]	-6,3 [ -9,8; -5,7]	-16 [ -18; -13]	-26 [ -36; -16]	-6,8 [ -16; -5,7]	-18 [ -30; -12]
Ширина зоны согласованности	28	9,9	21	29	8,9	21
Свободный член линии регрессии	9,3 [ 2,7; 16]	1,3 [ -2,6; 5,2]	7,2 [ 2,2; 12]	6,0 [ 2,3; 9,7]	0,69 [ -1,6; 2,9]	5,0 [ 2,2; 7,8]
Угловой коэффициент линии регрессии	-0,42 [ -0,58; -0,25]	-0,07 [ -0,17; 0,02]*	-0,33 [ -0,46; -0,2]	-0,35 [ -0,43; -0,27]	-0,06 [ -0,11; -0,02]	-0,28 [ -0,35; -0,22]
Максимальная ширина зоны согласованности	35,3	20,0	26,8	41,6	21,8	33,2

**Figure:**
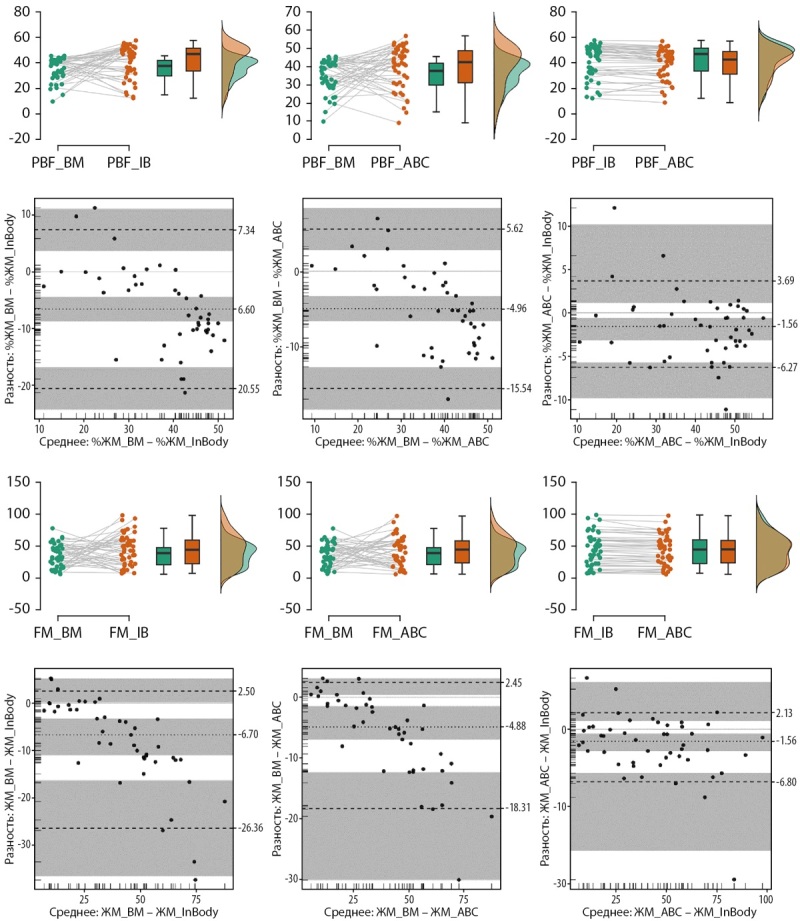
Рисунок 3. Графики Гарднера-Олтмена и Блэнда-Олтмена для значений жировой массы тела (FM), и ее доли (PBF), определенных в ходе УЗ-сканирования (BM), и различных биоимпедансных анализаторов (InBody770 и АВС-02). ССС — коэффициент конкордантной корреляции Лина. Пунктирные линии — среднее значение признака для пары методов и границы согласованности, серые области — 95%ДИ для среднего и границ согласованности.

Был проведен анализ эквивалентности для показателей относительного и абсолютного значения жировой массы, определенных разными методами (рис. 4). Статистическая эквивалентность подтверждена для %ЖМ и ЖМ, определенных на разных БИА анализаторах.

**Figure:**
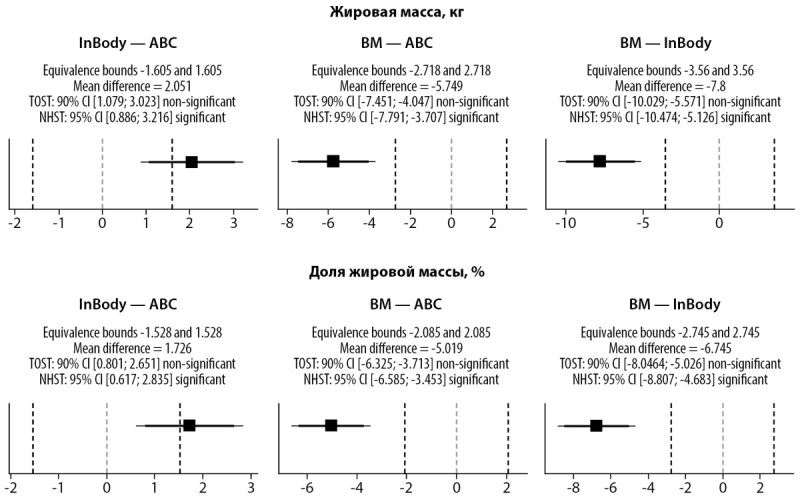
Рисунок 4. Статистический анализ на эквивалентность исследованных методов. Пунктирные линии — границы эквивалентности. Квадрат — разность средних и ее 90%ДИ.

В ходе проведения обследования нежелательных явлений зафиксировано не было. Все измерительных методы и процедуры являются неинвазивными и разрешены к применению с детского возраста. Проведение данных процедур не сопровождалось ухудшением самочувствия или другими жалобами со стороны обследованных добровольцев.

## ОБСУЖДЕНИЕ

Эпидемиологические исследования распространенности ожирения и избыточной массы тела, проведенные на территории РФ с использованием отечественного оборудования для импедансометрии, позволяют заключить, что на популяционном уровне 20% мужчин и 30% женщин имеют ожирение, а доля людей с ожирением увеличивается в старших возрастных группах [18]. Высокая доля ожирения и избыточной массы тела среди взрослого населения, а также необходимость сравнения результатов, полученных на разном оборудовании, стимулируют исследования в области методов, пригодных для массовых и полевых обследований гетерогенных групп. Вопрос согласованности оценок состава тела, получаемых с применением разных косвенных методов или разных приборов, не теряет своей актуальности [1][19][20]. Самым популярным методом косвенной оценки состава тела является БИА, однако ограничения данного метода затрудняют получение корректных данных у людей с некоторыми коморбидными ожирению заболеваниями и состояниями. В качестве альтернативы БИА в мире развивается направление УЗ-диагностики состава тела, которое сочетает точность и высокую воспроизводимость результатов без ограничений БИА [2][4][20]. На сегодняшний день представлено одно исследование, посвященное сопоставлению данных о составе тела, получаемых с применением отечественного и зарубежного анализатора АВС «Медасс» и Tanita. В настоящем исследовании проведен анализ согласованности трех различных приборов в объединенной группе мужчин и женщин, характеризующийся широким диапазоном морфологических характеристик. На групповом уровне БИА анализаторы АВС-02 «Медасс» и 770InBody являются взаимозаменяемыми для оценки %ЖМ и ЖМ (рис. 1 и 3). Также для них отсутствует пропорциональное смещение оценок на всем диапазоне измеряемых величин (рис. 3, табл. 2). Оценки ЖМ на приборах АВС-02 «Медасс» и 770InBody показывают высокий уровень согласованности, что позволяет сравнивать ЖМ, полученные на данных приборах, а также объединять оценки ЖМ в одну базу для последующего анализа. Оценки скелетно-мышечной массы, полученные в паре АВС-ВМ, не могут быть использованы без корректировки на групповом уровне, но не индивидуальном (табл. 2).

Характер различий в парах БИА-BodyMetrix сходный. В парах БИА-BodyMetrix присутствует расхождение в оценке жировой массы тела и ее доли, и оно возрастает с увеличением массы тела обследованных (рис. 3). При снижении ИМТ и, как следствие, ЖМ, оценки, полученные с использованием биоимпедансометрии, оказываются ниже, чем в УЗИ. В обследованной нами выборке разница в оценке ЖМ у мужчин и женщин с морбидным ожирением достигает 40 кг (рис. 3). Наличие смещения оценок ЖМ и %ЖМ в зависимости от ИМТ обследованных уже было показано для когорты женщин [20]. Ранее было показано, что BodyMetrixTM обладает высокой точностью оценки компонентов состава тела не только у молодых и здоровых взрослых, но и у людей с избыточной массой тела и ожирением, однако, по сравнению с воздушной плетизмографией, занижает содержание жирового компонента и завышает количество безжировой массы тела [4]. Отметим, что толщины подкожного слоя жира и толщины мышц — исходные данные, которые фиксирует УЗ-сканер, — в сочетании с другими антропометрическими признаками могут быть использованы для разработки прогностических уравнений жировой массы тела, отличных от классических антропометрических аналитических формул, реализованных в ПО ультразвукового сканера [18][22]. Сравнение УЗИ, БИА и воздушной плетизмографии (ВП) выявило высокую корреляционную связь между УЗИ и БИА (r=0,86), УЗИ и ВП (r=0,87) [21], сходные данные были получены и в нашем исследовании. Другое исследовании показало отсутствие между УЗИ и ВП значимой разницы, а также отсутствие систематического расхождения данных [22].

Поиск косвенного метода определения состава тела, который мог бы использоваться в широком диапазоне изменчивости морфологических показателей с точностью, сравнимой с лабораторными методами, привел к разработке методик, сочетающих в себе биоимпедансометрию и данные о толщинах кожно-жировых складок для расчета состава тела в рамках трехкомпонентной модели [23]. Вероятно, сочетание БИА и УЗИ может значительно улучшить точность определения состава тела в рамках трехкомпонентной модели. В перспективе необходимо проверить разработанные уравнения на новых группах женщин и мужчин, чтобы оценить работоспособность корректировок на новых данных и необходимости разработки уравнений для каждого пола.

## Клиническая значимость результатов

Показана возможность прямого сопоставления абсолютного значения жировой массы тела, полученного на разных приборах АВС-02 «Медасс» и 770InBody, на всем диапазоне значений ИМТ у совершеннолетних. Таким образом, референсные данные по половозрастным нормам, разработанные в масштабном популяционном исследовании на территории РФ для АВС «Медасс», могут быть использованы для данных о ЖМ, получаемых на 770InBody.

## Ограничения исследования

К ограничениям исследования относится небольшая численность обследованных мужчин и добровольцев с недостатком массы тела.

## Направления дальнейших исследований

Низкая согласованность оценок БИА-BodyMetrix, особенно у пациентов с морбидным ожирением, требует разработки процедуры коррекции данных, получаемых в УЗ-сканировании, с соответствующим контролем референсным методом.

## ЗАКЛЮЧЕНИЕ

Показан высокий уровень согласованности и статистическая эквивалентность двух БИА анализаторов отечественного производства АВС-02 «Медасс» и корейского 770InBody для показателей абсолютного и относительного количества жировой массы тела при использовании тетраполярной и полисегментной схемы соответственно. Прямое сравнение и/или объединение данных о составе тела, полученных с применением УЗ-сканера BodyMetrix и формул Джексона-Поллока по семи складкам, с данными БИА не рекомендуется, ввиду низкого уровня согласованности и пропорционального смещения оценок.

## ДОПОЛНИТЕЛЬНАЯ ИНФОРМАЦИЯ

Источники финансирования. Публикация подготовлена в рамках Государственного задания «Механизмы дезадаптации двухуровневой системы регуляции аппетита при экзогенно-конституциональном ожирении с множественными осложнениями и способы ее коррекции», Рег. № НИОКТР 122012100180-0.

Конфликт интересов. Трошина Е.А. — член редакционной коллегии журнала «Проблемы эндокринологии».

Участие авторов. Все авторы одобрили финальную версию статьи перед публикацией, выразили согласие нести ответственность за все аспекты работы, подразумевающую надлежащее изучение и решение вопросов, связанных с точностью или добросовестностью любой части работы.
